# Research on automatic classification technology of kidney tumor and normal kidney tissue based on computed tomography radiomics

**DOI:** 10.3389/fonc.2023.1013085

**Published:** 2023-02-24

**Authors:** Yunfei Li, Xinrui Gao, Xuemei Tang, Sheng Lin, Haowen Pang

**Affiliations:** Department of Oncology, The Affiliated Hospital of Southwest Medical University, Luzhou, China

**Keywords:** computed tomography (CT), radiomics, kidney, kidney tumor, automatic classification

## Abstract

**Purpose:**

By using a radiomics-based approach, multiple radiomics features can be extracted from regions of interest in computed tomography (CT) images, which may be applied to automatically classify kidney tumors and normal kidney tissues. The study proposes a method based on CT radiomics and aims to use extracted radiomics features to automatically classify of kidney tumors and normal kidney tissues and to establish an automatic classification model.

**Methods:**

CT data were retrieved from the 2019 Kidney and Kidney Tumor Segmentation Challenge (KiTS19) in The Cancer Imaging Archive (TCIA) open access database. Arterial phase-enhanced CT images from 210 cases were used to establish an automatic classification model. These CT images of patients were randomly divided into training (168 cases) and test (42 cases) sets. Furthermore, the radiomics features of gross tumor volume (GTV) and normal kidney tissues in the training set were extracted and screened, and a binary logistic regression model was established. For the test set, the radiomic features and cutoff value of P were consistent with the training set.

**Results:**

Three radiomics features were selected to establish the binary logistic regression model. The accuracy (ACC), sensitivity (SENS), specificity (SPEC), area under the curve (AUC), and Youden index of the training and test sets based on the CT radiomics classification model were all higher than 0.85.

**Conclusion:**

The automatic classification model of kidney tumors and normal kidney tissues based on CT radiomics exhibited good classification ability. Kidney tumors could be distinguished from normal kidney tissues. This study may complement automated tumor delineation techniques and warrants further research.

## Introduction

Radiomics refers to the high-throughput extraction of a large amount of information from medical images, such as computed tomography (CT), magnetic resonance imaging (MRI), and positron emission computed tomography (PET), to extract features and establish models. In general, visual image information is converted into digital feature variables for quantitative research. Extensive research and analysis of massive image data information can assist doctors in providing a more accurate diagnosis of a patient’s condition (1–3). Compared to biopsy, radiomics has the technical advantage of obtaining non-invasive and repeatable radiological images, thus providing a safer and more way for conducting patient follow-ups and prognosis prediction. Radiomics method can also be used for pathological tumor classification and grading (4, 5). Traditional radiomics, which involves the extraction and screening of high-throughput features of regions of interest from medical images, is used primarily for the diagnosis of benign and malignant diseases, prognosis evaluation, and survival prediction (6–13). Radiomics combined with machine learning and deep learning performs well for differentiating, grading and staging kidney tumors (14–16).

Traditional classification methods using computed tomography (CT) images mainly rely on the pixel value of the image and less on other parameters; this makes it difficult to accurately distinguish the tumor area from the surrounding normal organs (17–22). However, radiomics-based methods can be used to extract more than 800 radiomics features from regions of interest in CT images. In the present study, we proposed a radiomics research method based on the automatic classification technology of radiomics which has the potential to supplement the deep learning automatic delineation technology. This study is the first to report such a method. Therefore, the purpose of this study is to establish a preliminary classification model based on CT radiomics to automatically classify kidney tumors and normal kidney tissues.

## Materials and methods

### Data collection

The study was reviewed and approved by Ethics Committee of the Affiliated Hospital of Southwest Medical University (18 January 2017, KY2021023). CT data were retrieved from the 2019 Kidney and Kidney Tumor Segmentation Challenge (KiTS19) (https://wiki.cancerimagingarchive.net/pages/viewpage.action?pageId=61081171) of The Cancer Imaging Archive (TCIA) open access database. Arterial phase-enhanced images of 210 patients from the database were used to establish an automatic classification model. The kidneys and tumors were already manually segmented. These CT images were randomly divided into a training set (168 cases) and test set (42 cases).

### Radiomics feature extraction

**Figure 1:**
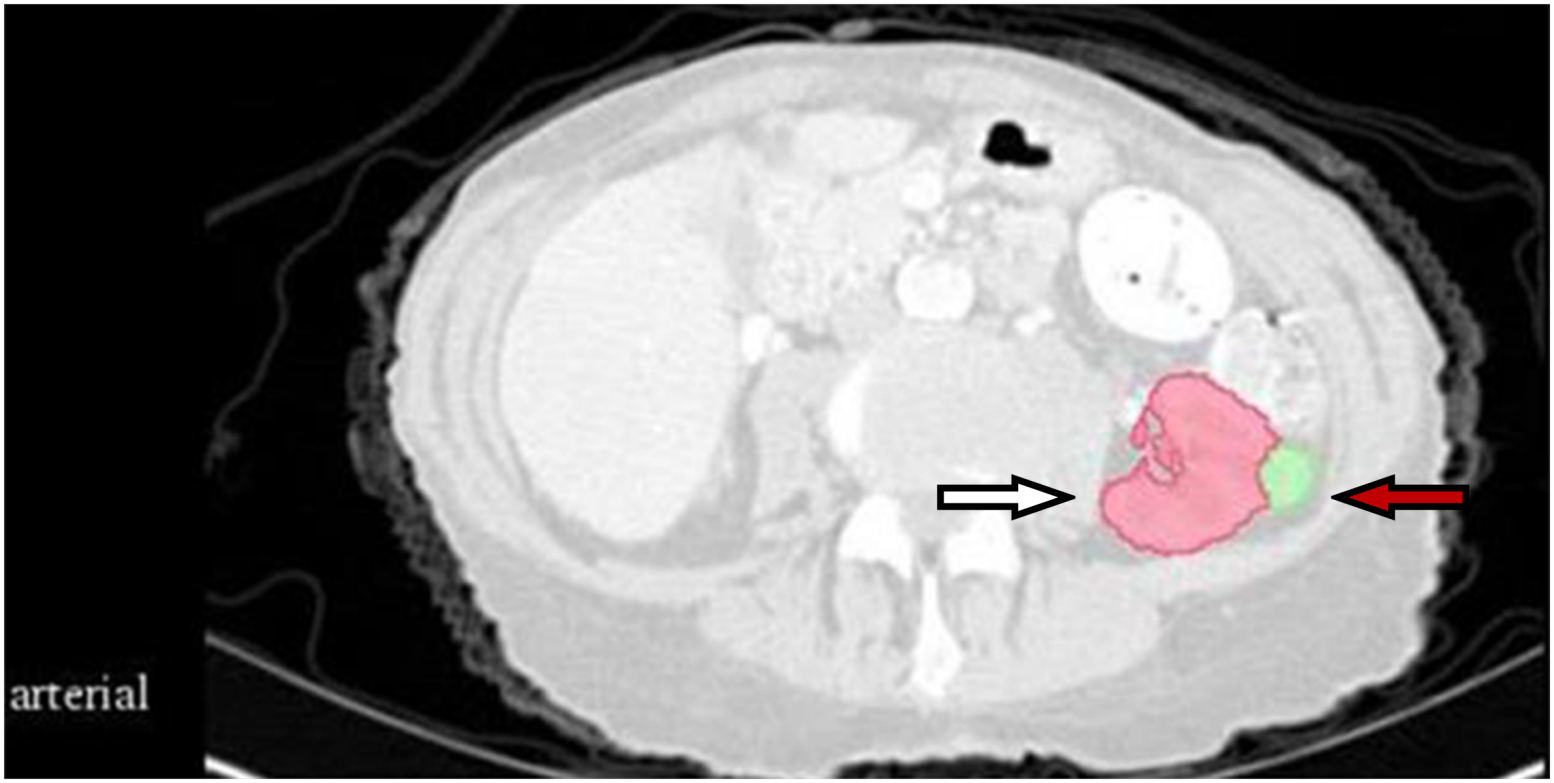
CT section of one patient in the training set. The white arrow indicates normal kidney tissue, and the red arrow indicates gross tumor volume.

The CT images were preprocessed using wavelet-based methods. Before feature extraction, all images were resampled according to a voxel size of 1 × 1 × 1 mm^3^. The gross tumor volume (GTV) and normal kidney tissue were regarded as the regions of interest. Feature extraction was based on a three-dimensional (3D) slicer platform and performed using the pyradiomics package; the package is available at http://PyRadiomics.readthedocs.io/en/latest/ (last accessed on June 30, 2019). The eigenvalue data of all radiomic features were processed using z-score standardization. **Figure 1** shows a CT sectional view of a patient in the training set. The CT radiomic feature variables of the GTV and normal kidney tissue were extracted. A total of 837 radiomics features were extracted, including first-order statistics, gray level co-occurrence matrix (GLCM), gray level dependence matrix (GLDM), gray level run length matrix (GLRLM), gray level size zone matrix (GLSZM), and neighboring gray tone difference matrix (NGTDM). Shape features were removed in this study. First-order features describes single pixel or voxel within the ROI. GLCM defines different combination of gray levels of an image area. GLDM quantifies the gray level dependencies in an image. GLRLM provides information about runs of consecutive pixels with the same gray level. GLSZM quantifies gray level zones in an image. And wavelet-based features were transformed based on above features.

### Screening of radiomics features of the training set

#### (A) Univariate feature screening

By considering the radiomics features of the GTV and normal kidney tissue as independent variables and the GTV and normal kidney tissue as binary variables, the area under curve (AUC; defined as the area surrounded by the receiver operation characteristic (ROC) curve and the abscissa and ordinate axis) corresponding to each radiomics feature was calculated. The AUC ranged from 0.5 to 1. The closer the value is to 1.0, the higher the authenticity, whereas an AUC value equal to 0.5 indicates that the radiomics have no application value in the study. The ROC curve takes the false positive rate (FPR) as the abscissa and true positive rate (TPR) as the ordinate. The curve is mostly used for the evaluation of binary classification problems. Radiomics features with an AUC less than 0.7 were excluded after the univariate screening.

#### (B) LASSO logistic regression feature screening

The principle of least absolute shrinkage and selection operator (LASSO) regression is to compress the original eigenvalue coefficients; more specifically, it involves directly compressing the original small coefficients to 0 and treating the eigenvalue variables corresponding to these coefficients as nonsignificant variables. Such nonsignificant variables have little or no impact on the final classification results; thus, such variables can be directly discarded, which results in variable screening. LASSO logistic regression was conducted using the method of five-fold cross-validation method to select the radiomics features. In the LASSO regression analysis, the L1 regularization term is added based on the least-squares fit to improve the accuracy of the linear regression model. Its penalty function is the absolute value of the regression coefficient, which guarantees that the parameter estimation results equal to zero. Thus, it is helpful for feature selection. This study is a binary classification problem, and logistic regression analysis is a generalized linear model commonly used in binary classification or one-to-many classifications. It normalizes the response of simple linear regression to zero and one. Therefore, the linear regression in the LASSO regression model can be replaced by logistic regression to select the characteristics. The objective function of LASSO logistic regression optimization is as follows:


$$\underset{\omega ,b}{\min}\sum_{i=1}^{n}\log(\exp(-y_{i}(X_{i}^{T}\omega +b))+1)+\lambda ∥\omega {∥}_{1}$$

where n is the total number of samples; Xi is an m × n-size raw data (each sample has m eigenvalues); yi is the corresponding response value of each sample; ω is the linear regression coefficient; b is the cutoff value of linear regression; and λ is a nonnegative regularization parameter used to control the sparsity of regression coefficients.

The extracted radiomics features were input into the LASSO logistic regression model; subsequently, the lambda (λ) value with the smallest model deviation was calculated and the radiomics features were screened.

#### (C) Model collinearity detection

The variance expansion factor (VIF) of the independent variable of the logistic regression model was calculated after screening the variables using the LASSO logistic regression. The VIF measures the severity of multicollinearity in multiple regression models. This represents the ratio of the variance of the estimator of the regression coefficient to the variance when no linear correlation between the independent variables is assumed.

The VIF can be calculated as follows.


$$\text{VIF}=\frac{1}{1-R_{i}^{2}},$$

where Ri is the negative correlation coefficient of the regression analysis for other independent variables. The larger the VIF, the greater the possibility of collinearity between the independent variables. Generally, multicollinearity is assumed when the VIF value is greater than five; thus, removing the radiomics features with a VIF value greater than five is necessary.

### Model establishment

The final binary logistic regression model was established based on the final radiomics features (X):


$$\text{logitP}=\text{g}\left({\text{x}}_{\text{i}}\right),$$

P = 1/ ( 1 + exp(−logitP)),

where P is the probability that GTV is positive. The ROC curve of the model was plotted and its AUC value was calculated. The sensitivity (SENS) and specificity (SPEC) corresponding to each point on the ROC curve were used to calculate the point that maximized SENS + SPEC, which is the cutoff value of P.

### Model diagnosis

The accuracy (ACC), SENS, SPEC, AUC, and the Youden index of the model were used for evaluating the effectiveness of the model. The Youden index, also known as the correct index, was used to evaluate the authenticity of screening tests. It is calculated as Youden index=SENS+ SPEC -1.

### Model validation

**Figure 2:**
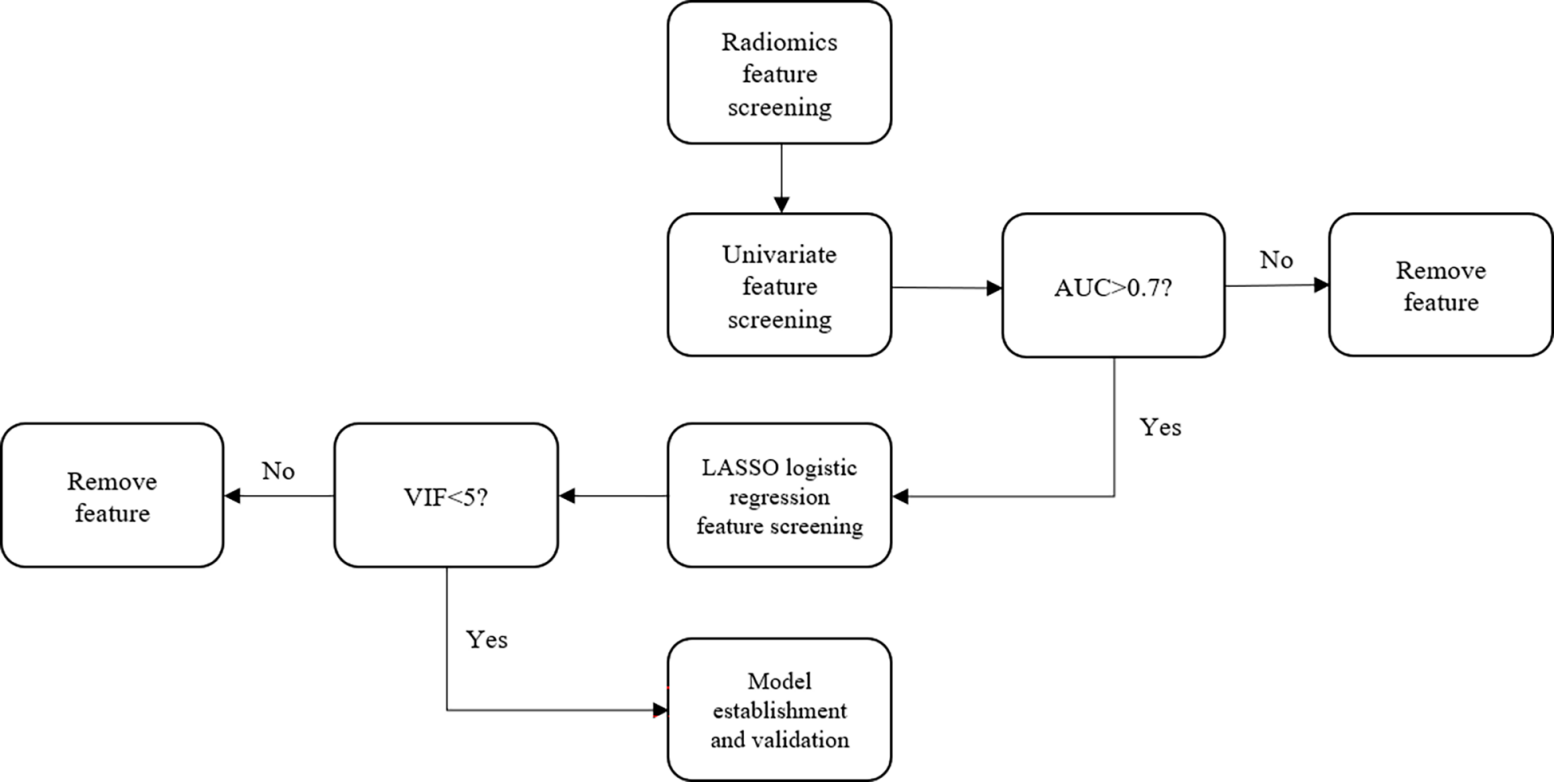
Flowchart of the proposed method.

For the test set, the radiomics features extracted concerning GTV and normal kidney tissue were consistent with those of the training set. The cutoff value of P in the test set is also consistent with the training set. If the GTV of the test set is positive, the ROC curve is drawn to calculate the AUC value. Thereafter, the ACC, SENS, SPEC, AUC, and Youden index of the test model were again calculated. The flowchart of the proposed method is shown in **Figure 2**.

All statistical analyses were performed using R software, version 4.1.2 (R Foundation for Statistical Computing, Vienna, Austria).

## Results

**Figure 3:**
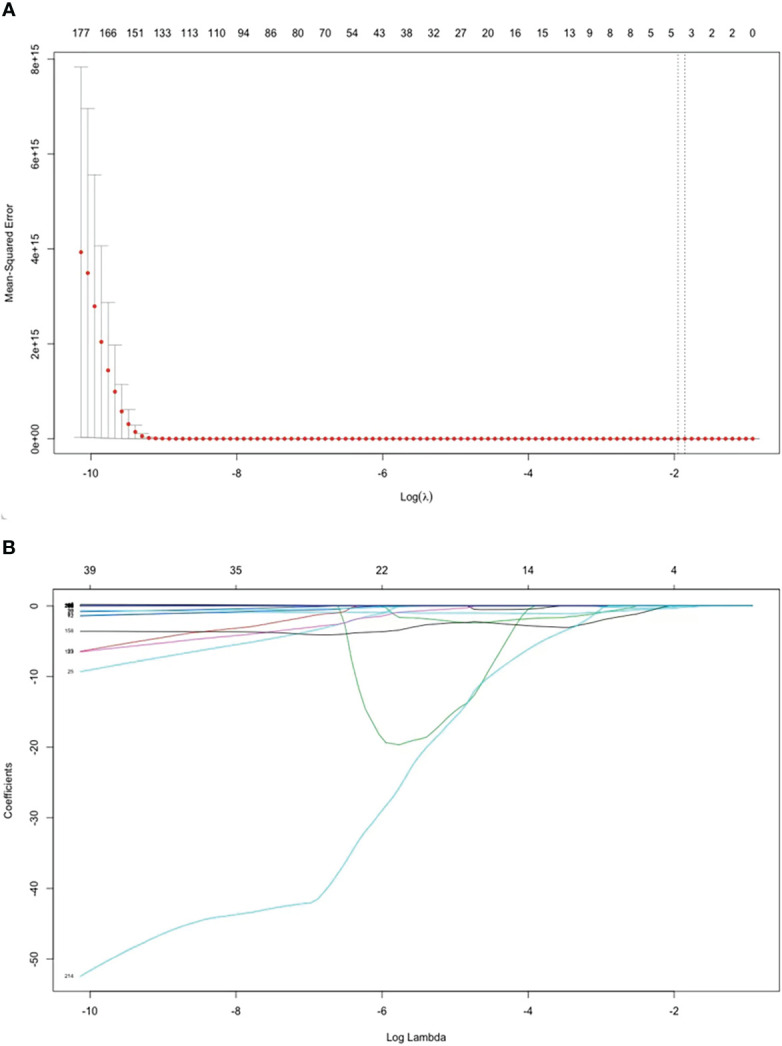
Features selected by the LASSO regression model. **(A)** Selection of the tuning parameter (*λ*) in the LASSO model *via* 5-fold cross-validation. The optimal *λ* are indicated by the dotted vertical lines, and a value of 0.1715 was selected. **(B)** LASSO coefficient profiles of 217 radiomics features. A coefficient profile plot was generated versus the selected log λ value using five-fold cross-validation. Three radiomics features with non-zero coefficients were selected.

Altogether, 837 radiomics features were extracted. After the univariate screening, 217 radiomic features were identified. Using LASSO logistic regression for variable screening, according to the calculation, the deviation of the model was the smallest when the minimum value was 0.1715; **Figure 3** shows the model deviation and lambda. Finally, three radiomics features were extracted: dependence entropy of the GLDM of the original (Feature 1), zone entropy of GLSZM of the wavelet-HLL (H = high-frequency band, L = low-frequency band; Feature 2), and gray level non-uniformity of GLSZM of the wavelet-LLL (Feature 3). **Table 1** lists the VIF values corresponding to these features. The VIFs were all less than five, which indicates that no multicollinearity existed among the three radiomics features.

**Table 1:** Model collinearity analysis.

Features	VIF
Feature 1	1.125322
Feature 2	1.052620
Feature 3	1.084162

The final binary logistic regression model was established based on the final radiomics features as follows.


logitP = −0.0156(Feature 1)−0.0523(Feature 2)−0.0004(Feature 3)

P = 1/ ( 1 + exp(−logitP)),

where P is the probability that GTV is positive. The cutoff value in this study was 0.4851. It was used as a radiomics marker to determine the tumor area and was equivalent to the critical point. If the detected value is greater than the cutoff value, it is the GTV; if the detected value is less than the cutoff value, it is the normal kidney tissue.

**Figure 4:**
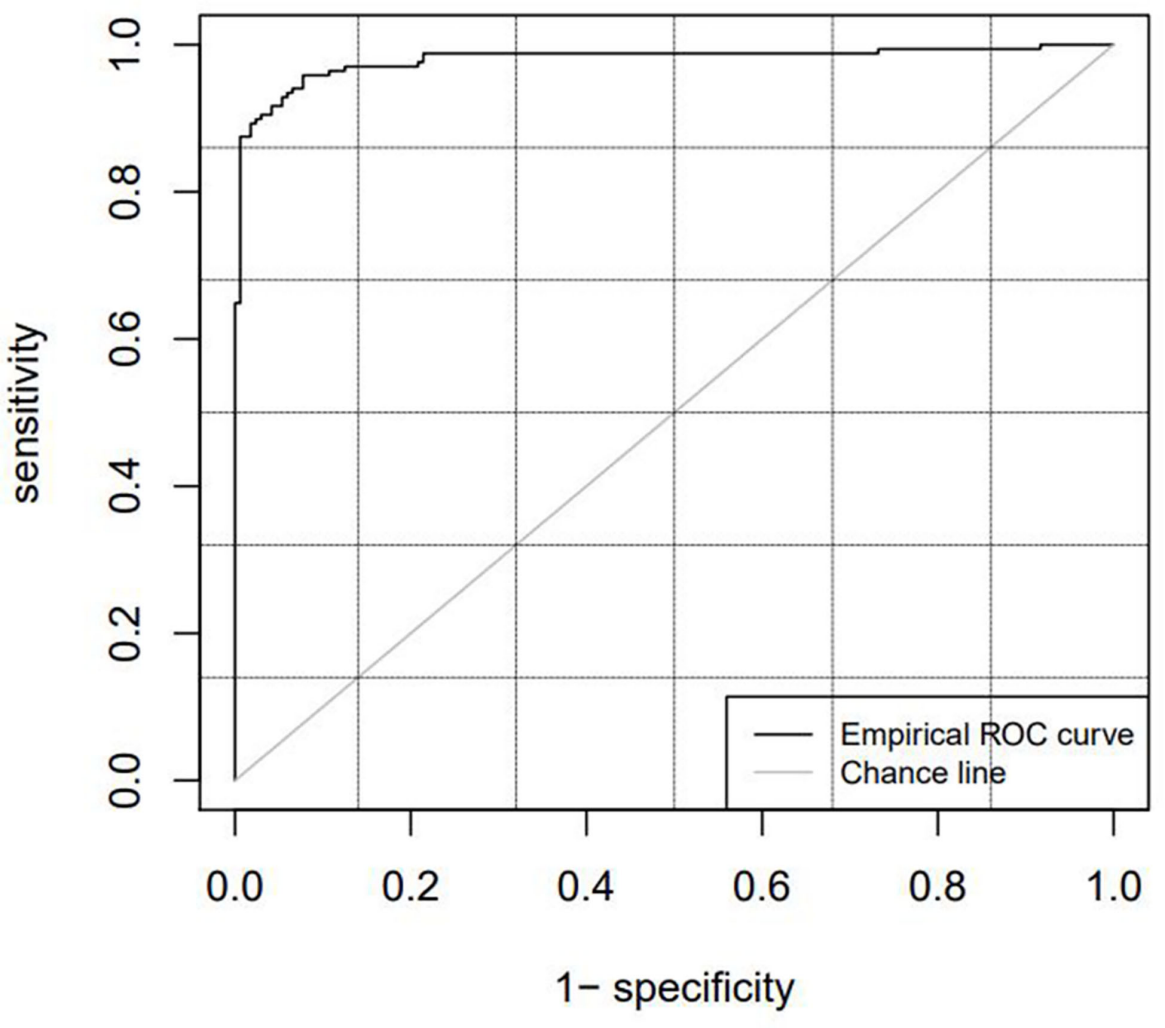
ROC curve of the training set.

**Table 2** lists the diagnostic parameters of the training set model, and **Figure 4** shows the ROC curve of the training set model.

**Table 2:** Training set model diagnosis parameters.

Cutoff (Predicted P value)	ACC	SENS	SPEC	AUC	Youden
0.4851	0.9405	0.9583	0.9226	0.9798	0.881

**Figure 5:**
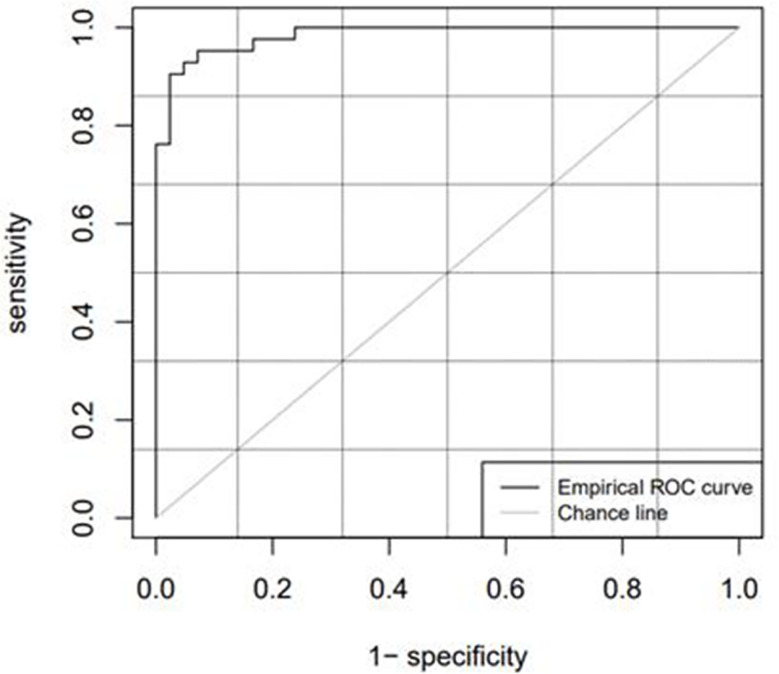
ROC curve of the test set.

**Table 3** lists the model diagnostic parameters of the test set, and **Figure 5** shows the ROC curve of the test set model.

**Table 3:** Test set model diagnosis parameters.

Cutoff(Predicted P value)	ACC	SENS	SPEC	AUC	Youden
0.4851	0.9286	0.9285	0.9285	0.9841	0.8571

## Discussion

Confirming the GTV is a challenge when treating a tumor. It tests the patience and proficiency of clinicians in relevant clinical knowledge. Furthermore, tumors of different shapes are difficult to delineate. A qualified clinician must have systematic learning and continuous practice to be competent in sketching. In this study, based on the CT radiomics method, the extracted CT radiomics features were used to automatically distinguish kidney tumor areas from normal kidney tissues.

The cutoff value, which is the critical point, was used as a radiomics marker for determining the GTV. If the detection value was greater than the cutoff value, it was considered positive; if the detection value was less than the cutoff value, it was considered negative. In this study, a positive detection value represented the kidney tumor part, and a negative value represented normal tissues and organs. The AUC values of the training and test sets obtained from the ROC curve were 0.9798 and 0.9841, respectively, which were significantly greater than 0.7. The ACC, SENS, SPEC, and Youden indices for the training and test sets based on the CT radiomics classification model were all greater than 0.85. This indicates that automatic classification technology based on radiomics had achieved good application results. Further studies need to extract only selected radiomic features from the GTV and kidney training sets instead of all radiomic features to improve efficiency.

In recent years, artificial intelligence technologies such as machine learning and neural networks have been widely used in the field of automatic tumor and organ delineation in radiotherapy, such as automatic segmentation technology based on convolutional neural network and automatic classification technology based on the U-NET model. These have greatly reduced clinician workload and increased productivity (23–26). Artificial intelligence models have achieved great success in the automatic delineation of organs, but the accuracy of the automatic delineation of tumor regions is still a problem. For future research, we can limit the region of interest to the entire kidney region, grid it, and extract only the radiomics features filtered through the training set to improve efficiency. A binary logistic regression model can be established based on the final radiomics features extracted from the training set, and the cutoff value calculated from the training set can be used as a radiomics marker for classifying tumor regions. When the P value is greater than the Cutoff value, the grid is considered to be a tumor region; when the P value is less than the Cutoff value, the grid is considered to be a normal organ. After clustering the tumor region or substituting P-values for pixel values, it is possible to automatically delineate the tumor region, which may be a supplement to the automatic delineation technology of deep learning.

In conclusion, automatic classification technology based on radiomics can be feasibly applied to distinguish between GTV and normal kidney tissue in patients. Nevertheless, our study has a few limitations. First, this study used a limited number of samples. Second, the establishment and optimization of the model were affected by the quality of the CT images and the accuracy of manual tumor segmentation. Third, different clinicians have a different understanding of GTV boundaries, and some CT images still have problems such as a fuzzy boundary of the tumor target area. Forth, the pre-malignant state and benign kidney lesions should be analyzed and differentiated. Thus, the data may have biases. For future clinical application, we plan to train and test CT image data of more patients with different tumors, to obtain better automatic classification results. If the technology is further matured and developed, it may reduce the risk of inaccurate drawing due to a lack of experience, save valuable processing time, and benefit doctors, patients, and radiotherapy technology.

Radiomics methods are generally used in the diagnosis of benign and malignant diseases, prognosis evaluation, and survival prediction. The preliminary method of automatic classification technology based on radiomics proposed in this study aims to enrich research on radiomics and may be to supplement to deep-learning automatic rendering technology to realize more accurate GTV determination. Future research should focus on different diseases and increase the number of samples to further improve the accuracy of this automatic classification technology.

## Conclusion

The automatic classification model of kidney tumors and normal kidney tissue based on CT radiomics exhibited good classification ability. Tumorous kidney tissues could be distinguished from normal kidneys, with these observations worthy of further study.

## Data availability statement

The original contributions presented in the study are included in the article/Supplementary Material. Further inquiries can be directed to the corresponding authors.

## Author contributions

HP and SL conceived the proposed concept and verified the underlying data. HP, YL, XG and XT took the lead in writing the manuscript. All authors provided critical feedback and helped shape the research, analysis, and manuscript. All authors contributed to the article and approved the submitted version.
